# Successful treatment of myeloid blast phase chronic myelogenous leukemia with the *JAK2* V617 F mutation by combination therapy with asciminib and ropeginterferon alfa-2b in an elderly patient

**DOI:** 10.1007/s12185-025-03994-2

**Published:** 2025-04-29

**Authors:** Satoko Oka, Yuina Akagi, Takaya Mituyoshi, Kazuo Ono

**Affiliations:** 1 Division of Hematology, Japanese Red Cross Society Wakayama Medical Center, Wakayama, Wakayama Japan; 2 Division of Pathology, Japanese Red Cross Society Wakayama Medical Center, Wakayama, Japan

**Keywords:** *BCR::ABL1*, *JAK2* V617 F mutation, Myeloid blast phase chronic myeloid leukemia (CML-myeloid BP), Asciminib, Ropeginterferon alfa-2b

## Abstract

The co-occurrence of *JAK2* V617F mutations and the *BCR::ABL1* translocation in the same patient is rare, and the current standard treatment for aggressive myeloid blast phase chronic myeloid leukemia (CML-myeloid BP) with *JAK2* V617F mutations remains inadequate, particularly in transplant-ineligible patients. Asciminib, a first-in-class allosteric inhibitor of BCR::ABL1 kinase that specifically targets the ABL1 myristoyl pocket, has emerged as a novel alternative to standard tyrosine kinase inhibitor (TKI) therapy. Ropeginterferon alfa-2b (ropegIFNα2b) is a novel site-selective, monopegylated recombinant human IFN with long-term safety and efficacy in patients with polycythemia vera (PV). Here, we report a case of successful combination therapy with asciminib and ropegIFNα2b in a patient with CML-myeloid BP who had a long history of PV with *JAK2* V617F refractory to induction chemotherapy with several TKIs. The combination of asciminib and ropegIFNα2b is a promising new treatment option for these patients.

## Introduction

The co-occurrence of the *BCR::ABL1* translocation and *JAK2* V617 F mutation is very rare, with fewer than 50 cases being reported in the literature [1–3]. The clonal composition of myeloproliferative neoplasms (MPN) harboring both *JAK2* V617 F and *BCR::ABL1* [4, 5] and the outcomes of these patients are unclear and management remains challenging.

The outcomes of patients with CML-myeloid BP are dismal and there is currently no established chemotherapy regimen. Since combination therapy with TKI leads to significantly higher response rates and longer survival than TKI or chemotherapy alone [6, 7], TKI remains a key component of treatment for CML-myeloid BP. Asciminib, a novel allosteric BCR::ABL1 inhibitor, targets the myristoyl binding pocket, with promising responses being reported in third-line treatment for chronic phase CML (CML-CP) after > 2 previous cycles of TKI [8]. The efficacy of asciminib in a Philadelphia chromosome (Ph)-positive acute lymphoblastic leukemia or blast phase CML (CML-BP) mouse model was recently demonstrated [9].

RopegIFNα2b is a novel site-selective, monopegylated recombinant human IFN that has been approved in Japan for patients with PV. The long-term safety and efficacy of ropegIFNα2b in patients with PV were recently reported [10]. Moreover, the combination of imatinib and ropegIFNα2b was shown to be safe and elicited a strong molecular response in patients with CML-CP not achieving deep molecular response (DMR) with imatinib alone [11].

We herein report a case of CML-myeloid BP with a long history of PV with *JAK2* V617 F refractory to induction chemotherapy with TKIs, including dasatinib, nilotinib, and bosutinib, which responded well to asciminib combined with ropegIFNα2b.

## Case presentation

A 70-year-old Japanese female was hospitalized with fever and general malaise.

**Fig. 1:**
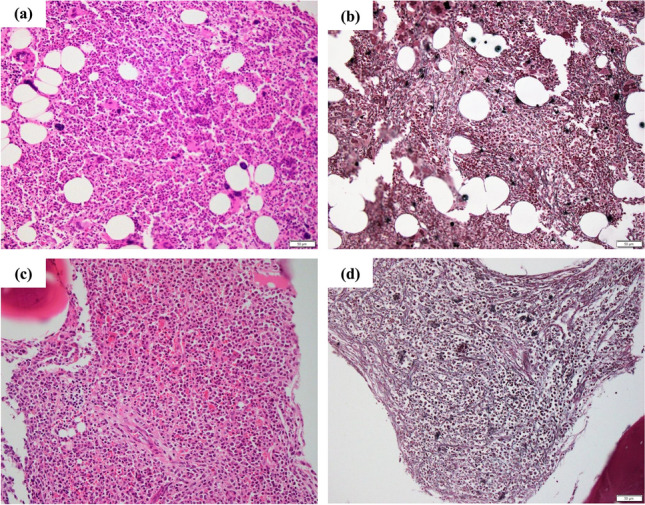
**a, b** A bone marrow (BM) examination revealed megakaryocytic hyperplasia (Hematoxylin and eosin, × 200) with grade1 fibrosis (Gomori, × 200) when the patient was diagnosed Ph-negative, *JAK2*-mutated PV. **c, d** A BM revealed hypercellularity (Hematoxylin and eosin, × 200) with grade1 fibrosis (Gomori, × 200) when the patient was diagnosed with CML-myeloid BP with *JAK2* V617 F mutation

**Fig. 2:**
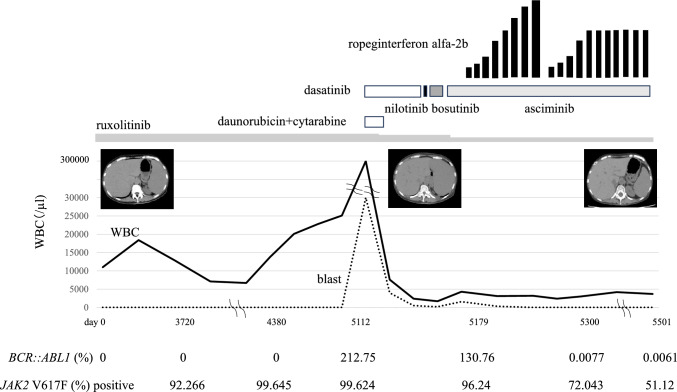
Clinical course

**Fig. 3:**
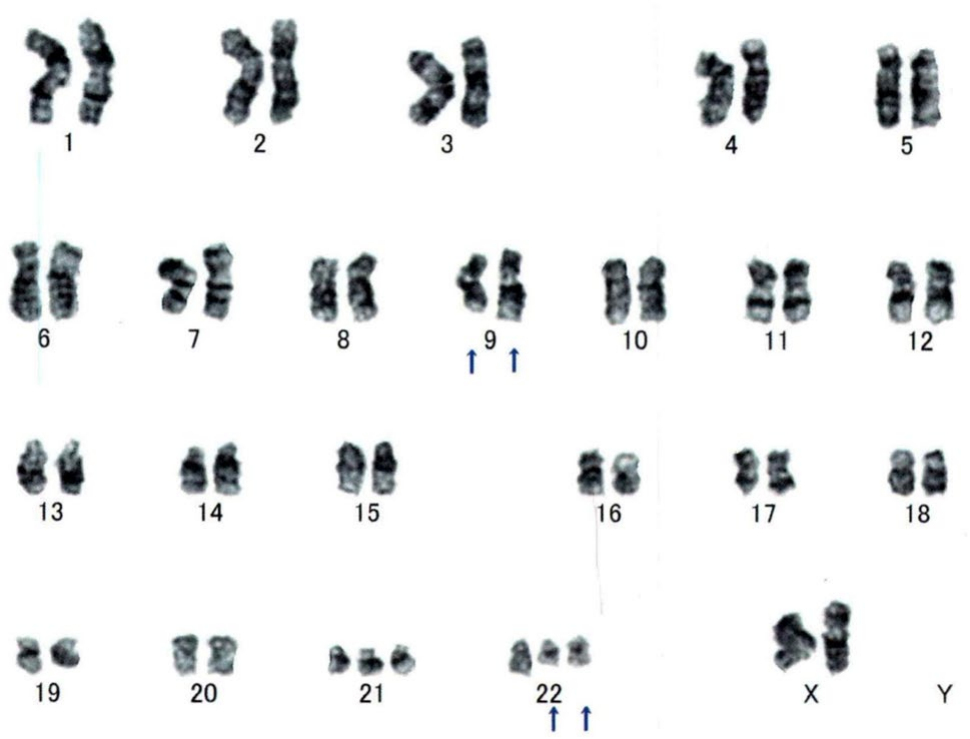
Karyotypic analyses showed 48, XX, inv(9)(p12q13), t(9;22)(q34.2:q11.2), + 21, + der(22)t(9;22) (20/20 cells)

The patient was diagnosed Ph-negative, *JAK2*-mutated PV when she was 56 years old. A bone marrow (BM) examination revealed megakaryocytic hyperplasia with grade1 fibrosis (Fig. 1a and b). She has been treated with ruxolitinib (10 mg twice daily) for 14 years. Laboratory tests (day 5112) showed a white blood cell count (WBC) of 270 × 10^9^/L, blast cell count of 75%, red blood cell count (RBC) of 239 × 10^4^/µL, hemoglobin (Hb) concentration of 75 g/L, hematocrit of 24.8%, and platelet count of 51 × 10^9^/L. Her serum level of lactase dehydrogenase (LDH) was 2274 U/L (normal range: 106–211 U/L). A flow cytometric analysis was positive for CD13, CD33, CD34, HLA-DR, and MPO. A chromosomal analysis revealed the Ph chromosome and a quantitative reverse transcriptase-polymerase chain reaction (RT-PCR) for *BCR::ABL1* fusion transcripts showed 212.75% for the *BCR::ABL1* fusion transcript of the p210 variant. Mutations in *BCR::ABL1* have not been detected by direct sequencing technique. At that time, *JAK2* V617 F remained positive with an allelic burden of 99.624%, while *MPL* and *CALR* were negative (Fig. 2). Karyotypic analyses showed 48, XX, inv (9) (p12q13), t(9;22)(q34.2:q11.2), + 21, + der(22)t(9;22) (20/20 cells) (Fig. 3). A BM examination revealed hypercellularity with grade 1 fibrosis (Fig. 1 c and d); however, BM aspiration failed due to a dry tap. Splenomegaly was observed on computed tomography (CT) scans (Fig. 2). Based on these findings, the patient was diagnosed with CML-myeloid BP.

Induction chemotherapy was initiated with daunorubicin 30 mg/m^2^ on days 1–5 + cytarabine 100 mg/m^2^ on days 1–7 with dasatinib 140 mg/day. Ruxolitinib was gradually reduced to 5 mg twice daily. After 3 weeks, laboratory data showed WBC of 2.4 × 10^9^/L, a blast cell count of 20%, RBC of 247 × 10^4^/µL, Hb concentration of 80 g/L, and platelet count of 68 × 10^9^/L. Her serum level of LDH was 1083 U/L. CTCAE grade 3 dasatinib-associated pleural effusion had developed. She was treated with diuretics and corticosteroid, and discontinued dasatinib. The patient was deemed intolerant, and nilotinib was introduced. However, she was intolerant due to the appearance of QTc prolongation. Bosutinib was introduced as third-line therapy. After 4 weeks, the patient developed fever, and laboratory data showed increases in WBC (4.3 × 10^9^/L), the blast cell count (37%), and LDH (1453 U/L). Asciminib 80 mg/day was introduced as fourth-line therapy. After 2 months (day 5179), laboratory data showed WBC of 3.1 × 10^9^/L, a blast cell count of 10%, RBC of 297 × 10^4^/µL, Hb concentration of 93 g/L, and platelet count of 102 × 10^9^/L. Her serum level of LDH was 980 U/L. RT-PCR for *BCR::ABL1* fusion transcripts revealed 130.76% for the *BCR::ABL1* fusion transcript of the p210 variant. At that time, *JAK2* V617 F remained positive with an allelic burden of 96.24%. RopegIFNα2b add-on therapy was initiated once every 2 weeks, at dose of 50 μg. The dose was increased by 50 μg every 2 weeks. Neutropenia (grade 2) occurred at a dose of 400 μg; however, no other severe adverse event was detected. RopegIFNα2b had been stopped until toxicity had resolved to ≤ grade1. Then, treatment as reintroduced with 50 μg every 2 weeks, and the dose was increased by 50 μg every 2 weeks. The treatment with 250 μg every 2 weeks has been continued. After 4 months (day 5300), laboratory data showed WBC of 3.2 × 10^9^/L, a blast cell count of 0%, RBC of 267 × 10^4^/µL, Hb concentration of 83 g/L, and platelet count of 50 × 10^9^/L. Her serum level of LDH was 140 U/L. After 6 months, RT-PCR for *BCR::ABL1* fusion transcripts showed 0.0077% for the *BCR::ABL1* fusion transcript of the p210 variant and *JAK2* V617 F remained positive with an allelic burden of 72.043%. Splenomegaly was improved on CT scans (Fig. 2). The patient refused allogenic SCT and has continued treatment with both drugs with good control of the disease 13 months after the diagnosis of CML-myeloid BP.

## Discussion

The co-occurrence of the *BCR::ABL1* translocation and *JAK2* V617 F mutation in the same patient is a rare event, ranging from 0.2 to 2.5% [12, 13]. These two genetic alternations may be identified simultaneously or *JAK2* mutations may be detected in the setting of previously diagnosed CML treated with TKI and the *BCR::ABL1* translocation ultimately developing in patients with a long history of *JAK2*-mutated MPN. The present case with a long history of *JAK2*-mutated PV subsequently developed CML-myeloid BP with the *BCR::ABL1* translocation. The clonal composition of MPN harboring both *JAK2* V617 F and *BCR::ABL1* has been discussed, with some studies favoring the presence of two independent clones and others supporting the hypothesis that the two genetic events occur in the same clone [4, 5]. In the present case, while it has not been confirmed through colony assays, *BCR::ABL1* transcript level at the time of CML diagnosis was approximately 200%, and this elevated level likely reflects the presence of double Ph. G-banding analysis showed that all 20 out of 20 examined cells harbored the Ph chromosome, and *JAK2* V617 F was detected with an almost 100% positivity rate. Although the methodologies for measuring gene mutation rates differ between *BCR::ABL1* and *JAK2* V617 F, this suggests that nearly all peripheral blood cells might have carried both *BCR::ABL1* and *JAK2* V617 F at the onset of CML. Furthermore, the fact that the *JAK2* V617 F allele burden did not decrease during the development of CML supports the notion that both genetic alternations coexisted within the same clone. These findings and clinical course strongly suggest the existence of a clonal population carrying both *BCR::ABL1* and *JAK2* V617 F mutations, and *JAK2* V617 F comes first and *BCR::ABL1* after at the same clone. Due to their relative rarity, the outcomes of patients carrying both *JAK2* V617 F and *BCR::ABL1* are unclear and management remains challenging. CML has been treated with different types of TKI and *JAK2*-mutated MPN with various therapies, including hydroxyurea (HU), IFN, anagrelide, and ruxolitinib; however, an optimal management strategy has yet to be established due to the paucity of follow-up data. Although promising outcomes with allogenic SCT were recently reported [12, 13], further data on SCT-ineligible patients are needed.

The treatment outcomes of patients with CML-BP remain dismal, with previous studies reporting median overall survival of 12 months or less [14]. It is extremely urgent to better understand the biology of disease progression and thereby illuminating the path of therapeutic strategies for effective management of patients with CML-BP. Possible mechanisms of therapeutic resistance against *BCR::ABL1*-dependent clones include point mutations in the *ABL1* kinase domain, *BCR::ABL1* splicing variants, *BCR::ABL1* overexpression, and altered pharmacokinetics by the ABC transporter [15]. The *BCR::ABL1* gene expression level has been considered to be associated with disease evolution in CML [16]. *BCR::ABL1* gene expression level can be increased by upregulation of transcript level or by amplification of *BCR::ABL1* fusion gene or by increase in number of *BCR::ABL1*-positive leukemic cells in the peripheral blood or BM. Overexpression of *BCR::ABL1* protein due to amplification of *BCR::ABL1* fusion gene was first identified in imatinib resistant CML cell lines [17]. In this case, RT-PCR for *BCR::ABL1* fusion transcripts revealed 212.75% and the patient developed rapidly progressing clinical symptoms after third TKI treatment. It is suggested that the overexpression of *BCR::ABL1* might be an early phenomenon in the establishment of TKI resistance and disease evolution in CML.

In a large retrospective study on CML-myeloid BP patients, combination therapy with TKI led to significantly higher response rates and longer survival than TKI or chemotherapy alone [6, 7]. TKI remains a key component of treatment for CML-myeloid BP. Asciminib, a first-in-class allosteric inhibitor of BCR::ABL1 kinase that specifically targets the ABL1 myristoyl pocket, has emerged as a novel alternative to standard TKI [8]. Its efficacy to overcome TKI resistance and enhance DMR and treatment-free remission rates with a non-overlapping toxicity profile as well as its effectiveness as a selective ABL kinase inhibitor, with a potentially more tolerable safety profile in patients with CML-CP, have recently been reported [18, 19]. Chatain et al. utilized a tetracycline-inducible p210 *BCR::ABL1* transgenic mouse model and demonstrated the efficacy of asciminib for Ph-positive acute lymphoblastic leukemia or CML-BP [9].

Recent studies demonstrated that IFN treatment for PV may reduce the allele burden of driver mutations, which suggests a disease-modifying effect that is not observed with purely symptomatic treatment using aspirin and HU [20]. RopegIFNα2b is a novel site-selective, monopegylated recombinant human IFN, and PROUD-PV and CONTINUATION-PV showed that it was effective for and tolerated well by patients with PV [21, 22]. Kirito et al. demonstrated the safety and efficacy of ropegIFNα2b over 36 months in Japanese patients with PV [10]. IFN was also used for CML before the TKI era and a subset of patients defined as “good responders” to IFN was functionally cured despite detectable residual molecular disease. Prior treatment with IFN was associated with a higher rate of a sustained major molecular response 6 months after discontinuation in the EURO-SKI study [23]. In a phase I study, the combination of imatinib and ropegIFNα2b was safe and elicited a strong molecular response in patients with CML-CP not achieving DMR with imatinib alone [11]. This case was CML-myeloid BP with the *JAK2* V617 F mutation, and the combination of asciminib and ropegIFNα2b achieved early and sustained major molecular response.

We herein described a case of CML-myeloid BP with a long history of PV with *JAK2* V617 F refractory to induction chemotherapy with TKIs, including dasatinib, nilotinib and bosutinib, which responded well to asciminib combined with ropegIFNα2b. The co-occurrence of *JAK2* V617 F mutations and the *BCR::ABL1* translocation in the same patient is rare and the current standard treatment for aggressive CML-myeloid BP with *JAK2* V617 F mutations, particularly in patients who are not eligible for SCT, remains inadequate. The combination of asciminib and ropegIFNα2b therapy has potential as a new and promising treatment option for these patients. 

## Data Availability

All data that support the findings of this study are available from the corresponding author upon reasonable request.
